# Murine SIGNR1 (CD209b) Contributes to the Clearance of Uropathogenic *Escherichia coli* During Urinary Tract Infections

**DOI:** 10.3389/fcimb.2019.00457

**Published:** 2020-01-10

**Authors:** Yingmiao Zhang, Song Zhang, Yingxia He, Ziyong Sun, Wentong Cai, Yin Lv, Lingyu Jiang, Qiao Li, Sizhe Zhu, Wenjin Li, Chenglin Ye, Bicong Wu, Ying Xue, Hongxiang Chen, Huahua Cai, Tie Chen

**Affiliations:** ^1^Department of Clinical Immunology, Tongji Hospital, Tongji Medical College, Huazhong University of Sciences and Technology, Wuhan, China; ^2^Department of Dermatology, Union Hospital, Tongji Medical College, Huazhong University of Science and Technology, Wuhan, China; ^3^Department of Clinical Laboratory, Tongji Hospital, Tongji Medical College, Huazhong University of Sciences and Technology, Wuhan, China; ^4^State Key Laboratory of Veterinary Biotechnology, Harbin Institute of Veterinary Medicine, Chinese Academy of Agricultural Sciences, Harbin, China

**Keywords:** uropathogenic *E. coli*, urinary tract infection, macrophage, DC-SIGN, SIGNR1

## Abstract

Uropathogenic *Escherichia coli* (UPEC), a Gram-negative bacterial pathogen, is a major causative agent of urinary tract infections (UTIs). However, the molecular mechanisms of how UPEC causes infections have not been determined. Recent studies indicated that certain enteric Gram-negative bacteria interact with and hijack innate immune receptors DC-SIGN (CD209a) and SIGNR1 (CD209b), often expressed by antigen-presenting cells (APCs), such as macrophages, leading to dissemination and infection. It was not known whether UPEC could utilize DC-SIGN receptors to promote its infection and dissemination similarly to the enteric pathogens. The results of this study reveal that UPEC interacts with CD209-expressing macrophages and transfectants. This interaction is inhibited by anti-CD209 antibody, indicating that CD209s are receptors for UPEC. Additionally, in contrast to the results of previous studies, mice lacking SIGNR1 are more susceptible to infection of this uropathogen, leading to prolonged bacterial persistence. Overall, the results of our study indicate that the innate immune receptor CD209s participate in the clearance of UPEC during UTIs.

## Introduction

The major causative agent of urinary tract infections (UTIs) is Gram-negative uropathogenic *Escherichia coli* (UPEC), which accounts for ~80% of community-acquired infections and 25% of nosocomial infections (Ronald, 2002). UTIs are one of the most common bacterial infections, with an annual incidence of 150 million cases worldwide (Stamm and Norrby, 2001). More than half of all women and some men will have at least one UTI in their lifetime (Foxman, 2002). Recent increases in the prevalence of multidrug-resistant uropathogenic strains, together with the lack of an effective vaccine against UPEC infections, have become worldwide concerns and present a substantial challenge to public health (O'Brien et al., 2016; Sanchez et al., 2016). A hallmark of such infections is high incidence of recurrence, which occurs in 20–30% patients (Foxman, 2002).

Bacterial ascension into the urinary tract may lead to infection. After reaching the bladder mucosa, UPEC can be internalized by superficial bladder epithelial cells (facet or umbrella cells) and can form intracellular bacterial communities (IBCs) or quiescent intracellular reservoirs (QIRs) that are resistant to phagocytosis by polymorphonuclear leukocytes (PMNs) and macrophages, and are resistant to antibiotic treatment (Schilling et al., 2002; Kerrn et al., 2005; Justice et al., 2006; Mysorekar and Hultgren, 2006; Horvath et al., 2011). Upon lysis of facet cells, the released UPEC can bind and invade neighboring bladder cells to start the cycle anew (Anderson et al., 2003; Justice et al., 2004), suggesting that the UPEC utilization of facet cells provides a shelter, allowing persistence in the bladder. Antigen presenting cells (APCs), such as dendritic cells and macrophages, play essential roles in the host immune response to UTIs (Mora-Bau et al., 2015). In addition to antigen presentation, macrophages are also important sources of pro-inflammatory cytokines that modulate inflammatory responses during UTIs (Abraham and Miao, 2015; Schaale et al., 2016; Waldhuber et al., 2016). Macrophages and neutrophils work together as inducers and effectors upon bacterial infections, and the cooperation between tissue resident macrophages and inflammation-recruited macrophages is involved in neutrophil migration (Schiwon et al., 2014). CD14 is involved in immune cell migration and differential cytokine production in macrophages, and studies by Carey et al. demonstrated a protective role of CD14 expressed by monocytes/macrophages in UTIs (Carey et al., 2016). Current studies have shown that UPEC can subvert the host immune response by suppression of early urothelial cytokine production and inhibition of neutrophil recruitment and function (Klumpp et al., 2001; Billips et al., 2007; Loughman and Hunstad, 2011). Interestingly, it is reported that UPEC can subvert macrophage anti-microbial pathways and survive inside macrophages up to 24 h (Bokil et al., 2011; Stocks et al., 2019). However, the molecular mechanisms of the development of persistent UPEC infections are not fully understood.

Recent studies indicate that several Gram-negative bacteria, including *Salmonella* Typhimurium (*S*. Typhimurium), *Yersinia pseudotuberculosis* (*Y. pseudotuberculosis*) and *Yersinia pestis* (*Y. pestis*), are able to interact with DC-SIGN (CD209s), a C-type lectin and an innate immune receptor expressed by APCs, to hijack the APCs for host dissemination and infection (He et al., 2019; Yang et al., 2019; Ye et al., 2019). These studies were informed by the mechanism of HIV infection of APCs. HIV binds the human DC-SIGN receptor on APCs and hijacks infected DCs as Trojan horses to promote viral dissemination to target cells such as CD4 lymphocytes in lymph nodes (Geijtenbeek et al., 2000; Engering et al., 2002; McDonald et al., 2003). In addition, Helaine et al. found that the phagocytosed *S*. Typhimurium was able to form persisters inside macrophages, which provide a reservoir for relapsing infection (Helaine et al., 2014; Rycroft et al., 2018). Consequently, macrophages that are supposed to be warriors of innate immunity are subverted by pathogens to promote their persistence in hosts. Understanding the details of these macrophage interactions may provide a better understanding of the pathogenesis of intracellular pathogens.

The lipopolysaccharides (LPS) of many Gram-negative bacterial pathogens promote resistance to serum killing and/or phagocytosis (Burns and Hull, 1998; Cortes et al., 2002; Murray et al., 2003). Over 70% of the cell surface of Gram-negative bacteria is occupied by LPS, which consists of lipid A, the oligosaccharide core (LPS core), and O-antigen. Our previous studies show that N-acetylglucosamine (GlcNAc) sugar residues within the LPS core of several Gram-negative bacteria are exposed by loss of O-antigen, which helps in the targeting of CD209s by these pathogens (Zhang et al., 2006). The host dissemination and infection of some pathogens can be significantly reduced by inhibition of such receptor-ligand interactions with an oligosaccharide or its analogs (He et al., 2019; Yang et al., 2019; Ye et al., 2019), indicating that inhibition of LPS core-CD209s interaction may serve as a treatment or prevention strategy of infections caused by these pathogens.

In this study, we examined whether the Gram-negative UPEC can interact with CD209s to promote host infection and persistence during urinary tract infections.

## Materials and Methods

### Bacterial Strains and Cell Lines

The bacterial strains and cell lines used in this study are listed in Table 1. Human uropathogenic *Escherichia coli* (UPEC) cystitis isolate UTI89 and pyelonephritis strain CFT073 were obtained from Harbin Institute of Veterinary Medicine, Chinese Academy of Agricultural Sciences, Harbin, China. UTI89-GFP is UTI89 transformed with the green fluorescent protein-expressing plasmid pAcGFP1 (Clontech, CS, USA). UTI89-O^+^ and CFT073-O^+^ were constructed by transformation with the pAY100.1 plasmid, which carries the genes necessary for *Yersinia enterocolitica* O-antigen expression (Oyston et al., 2003). UPEC1-UPEC4 strains were clinically isolated from urine samples from the Department of Clinical Laboratory of Tongji Hospital. *E. coli* K-12 strain CS180 produces a rough LPS lacking O-antigen (Schnaitman and Klena, 1993). CS1861 is a derivative of CS180 harboring pSS37, a plasmid containing all of the genes for O-antigen expression from *Shigella dysenteriae* 1 (Klena et al., 1992; Klena and Schnaitman, 1993). *Y. pseudotuberculosis* Y1 is a serotype O:1a strain lacking the virulence plasmid (pYV). This strain was obtained from the Centers for Disease Control (GA, USA) and was used previously as a positive invasion control (Chen et al., 1995), since this bacterium invades almost all epithelial cell lines via an invasin-integrin interaction (Isberg and Leong, 1990).

**Table 1:** Bacterial strains and cell lines used in this study.

**Strains**	**Genotypes(phenotypes)**	**References**
Uropathogenic *E. coli*		
UTI89	Human UPEC cystitis isolate	Mulvey et al., 1998
UTI89-GFP	UTI89 carries pAcGFP1 plasmid	This study
UTI89-O^+^	UTI89 carries pAY100.1 plasmid expressing an O-antigen from *Y. enterocolitica* serotype O:3	This study
CFT073	Pyelonephritis strain	Cai et al., 2017
CFT073-O^+^	carries pAY100.1 plasmid expressing an O-antigen from *Y. enterocolitica* serotype O:3	This study
UPEC1	Clinical isolate	This study
UPEC2	Clinical isolate	This study
UPEC3	Clinical isolate	This study
UPEC4	Clinical isolate	This study
*E. coli* K-12		
CS180	Contains core LPS but lacks O-antigen (rough)	Klena et al., 1992; Klena and Schnaitman, 1993
CS1861	CS180 expressing O-antigen (smooth)	Klena et al., 1992; Klena and Schnaitman, 1993
*Y. pseudotuberculosis*		
Y1	O:1a, wild-type expressing invasin but with pYV plasmid naturally cured	Chen et al., 1995
**Cell lines**	**Characteristics**	
CHO-NEO cells	Control cell line, which expresses the neomycin resistance gene only	
CHO-mSIGNR1 cells	Generated by transfecting CHO cells with mouse SIGNR1 cDNA for stable surface expression	
CHO-hDC-SIGN cells	Generated by transfecting CHO cells with human DC-SIGN cDNA for stable surface expression	
Peritoneal macrophages	Isolated from mouse peritoneal cavity	

CHO-mSIGNR1 and CHO-hDC-SIGN were generated by transfecting CHO cells (Type Culture Collection, Chinese Academy of Sciences, Shanghai, China) with mouse SIGNR1 cDNA and human DC-SIGN cDNA, respectively, followed by selection for stable surface expression as originally described (Takahara et al., 2004). CHO-NEO is used as a control cell line that expresses only the neomycin resistance gene.

### Mice

C57BL/6 wild-type (WT) mice were purchased from Wuhan University Animal Center. SIGNR1 knockout (SIGNR1^−/−^) mice on a C57BL/6 background were kindly provided by the Consortium for Functional Glycomics (http://www.functionalglycomics.org) and bred in the animal facility of Tongji Hospital. The WT and SIGNR1^−/−^ mice used were matched in age and weight. After obtaining from the vendor, the WT mice were housed in pathogen-free conditions in the animal facility for more than 2 weeks before the experiment. All mice were treated in direct accordance with guidelines drafted by the Animal Care Committees of Tongji Hospital.

### Isolation of Mouse Peritoneal Macrophages

After mice were euthanized, their abdomens were immediately exposed, cleaned with 70% ethanol and opened with scissors. Next, 5 ml of RPMI media was injected into the intraperitoneal cavity. Abdomens were gently massaged for 2 min and the lavage fluid was then collected. The suspension containing macrophages was seeded in flasks and placed in a CO_2_ incubator for 2 h. The cell layers were washed three times to remove non-adherent cells. Macrophages were then removed from the plastic surface by incubating with citrate saline and re-seeded for interaction assays.

### Invasion Assays

The invasion assays were carried out as described previously (Chen et al., 1997, 2001). Briefly, host cells were plated in 24-well plates. Cells were suspended in RPMI media with 2% fetal calf serum (FCS) at a concentration of 4×10^5^ cells/ml, and 0.5 ml aliquots of cell suspensions were added to 24-well plates. After addition of 50 μl of bacterial suspension at a concentration of 2×10^7^ colony forming units (CFUs) /ml (2×10^6^ CFUs/ml for *Y. pseudotuberculosis* Y1 due to its high level of invasiveness), the cells were incubated for 2.5 h (2 h for macrophages) at 37°C in the presence of 5% CO_2_. The cell monolayers were then washed three times with phosphate-buffered saline (PBS). Gentamycin, which kills extracellular bacteria but cannot penetrate into host cells, was added into each well to a final concentration of 100 μg/ml, and the cultures were incubated at 37°C for 60 min. Cells were washed twice to remove the antibiotic, suspended in PBS containing 0.2% Triton X-100 (Sigma-Aldrich, MO, USA), diluted, and plated on Luria-Bertani (LB)-agar plates. The level of internalization of bacteria in host cells was calculated by determining the CFUs recovered from lysed cells.

For the inhibition assay, Mannan (500 μg/ml), anti-hDC-SIGN antibody (5 μg/ml), and anti-mSIGNR1 antibody (5 μg/ml) were added 20 min prior to the addition of bacteria. The concentrations used were determined based on our preliminary data and were selected because at these concentrations, the compounds did not affect the survival of bacteria or host cells, as previously shown (Zhang et al., 2006, 2008).

### LPS Extraction and Staining

For further analysis of the O-antigen expression pattern of UPEC strains, LPS samples were extracted and purified from UPEC strains using an LPS extraction kit (iNtRON Biotechnology, Korea), performed according to the manufacturer's instructions. The *E. coli* strains CS180 and CS1861, which show rough LPS (without O-antigen) and smooth LPS (with O-antigen), respectively, were used as control strains. After purification, the LPS samples were analyzed using a PAGE silver staining kit (Solarbio Science & Technology, Beijing, China). After a series of washes, incubation, and termination, the signals were visualized using a Bio-Rad gel imager (Hercules, CA, USA).

### Intraperitoneal Infections

WT and SIGNR1^−/−^ mice (five mice/group) at 8 weeks of age were intraperitoneally injected with 10^6^ CFUs of UTI89 suspended in 100 μl PBS. All mice were monitored according to ethics requirements. After 24 h of infection, the mice were sacrificed and livers, spleens, and mesenteric lymph nodes (MLNs) were collected, weighed, and washed in PBS with Gentamycin (100 μg/ml) to remove bacteria that had not invaded tissues. Tissues were washed three times to remove the antibiotic, and then homogenized in 1 ml 1% Triton X-100. Dilutions were spread on LB-agar plates and incubated overnight before CFUs were counted to determine CFUs per organ.

### Mouse and UTI Models

Six- to eight-week-old mice were catheterized for inoculation of 10^8^ CFUs of UTI89 suspended in 30 μl PBS under sodium pentobarbital anesthesia (Chan et al., 2013). Control experiments were performed with application of 30 μl PBS. At the indicated times after infection, mice were individually induced to urinate by applying gentle pressure to the skin just below the occiput. The clean-catch urine samples were collected into sterile Eppendorf tubes (Eppendorf, Hamburg, Germany), and bacterial titers were determined by plating serial dilutions of the material onto LB-agar plates. Dilutions were also examined under microscopy for polymorphonuclear leukocyte (PMN) levels with a hemocytometer (Bioleaf Biotech Co., Ltd, Shanghai, China). Bacterial titers in the bladder were determined by homogenizing the tissue in 1 ml of sterile PBS containing 0.5% Triton X-100, and plating serial dilutions of the homogenate onto LB-agar plates. Mice were sacrificed by cervical dislocation under anesthesia, and their bladders were aseptically removed and frozen before sectioning.

### Histopathology and Immunofluorescence

Bladder tissues were aseptically harvested from mice at the indicated time points, and 4% paraformaldehyde fixed tissues were embedded in paraffin, sectioned, and stained with hemotoxylin and eosin and Masson's trichrome. Bladder inflammatory scores over the total area of each section were determined by criteria as previously described (Hopkins et al., 1998).

Peritoneal macrophages collected from WT and SIGNR1^−/−^ mice were cultured in 24-well plates (1×10^5^ cells per well, in a volume of 1 ml). After incubation with 10^6^ CFUs of UTI89-GFP for 2 h, the cell layers were washed three times with PBS to remove unbound bacteria. Cells were fixed in cold methanol for 10 min and incubated for 1 h in 1% BSA (Sigma-Aldrich, MO, USA) and 0.3% Triton X-100 in PBS to block non-specific binding and permeabilize the cells. The cells were then incubated for 1 h at room temperature with a primary rabbit IgG antibody (Sino Biological Inc., Beijing, China) directed against mouse SIGNR1. Primary antibodies were detected with a goat anti–rabbit IgG-conjugated with Alexa Fluor 594 (Invitrogen, CA, USA). The cell-associated bacteria were quantified by counting cells and bacteria that attached to or inside macrophages in three fields under microscope, and bacterial count per 100 cells was calculated.

For immunofluorescence analysis, frozen bladder sections were cut and fixed in acetone at −20°C for 10 min. All sections were hydrated and blocked in 1% BSA, 0.3% Triton X-100 in PBS. After incubation with primary antibodies to detect against mouse SIGNR1 (Sino Biological Inc. Beijing, China) and *E. coli* (Abcam, MA, USA), incubation with secondary antibodies, and associated washes, slides were stained with bis-benzimide (Sigma, MO, USA). Stained tissues were examined by epifluorescence microscopy on a ZEISS Axioskop 2 MOT Plus microscope.

### ELISA

Bladder tissue was harvested at indicated time points and stored at−80 °C until all sampled had been collected. For measuring interleukin-6 (IL6), tumor necrosis factor (TNF)-α and interleukin-10 (IL10) expression in bladder homogenates, enzyme-linked immunosorbent assay (ELISA) Kits (Shanghai Enzyme-linked Biotechnology Co., Ltd. Shanghai, China) using matched antibody pairs were used according to the manufacturer's instructions.

### Statistical Analyses

Statistical tests were obtained using the Prism software version 6 (Graph Pad, San Diego, CA, USA). Statistically significant differences between two groups were determined by non-parametric *t*-test and comparison among three or more groups was obtained by one-way or two-way analysis of variance (ANOVA) with multiple comparisons test. Additionally, *p*-values were deemed significant if < 0.05. Data are presented as mean values ± standard error of mean (SEM).

## Results

### Macrophages From SIGNR1^−/−^ Mice Have Reduced Phagocytosis of Uropathogenic *E. coli*

**Figure 1:**
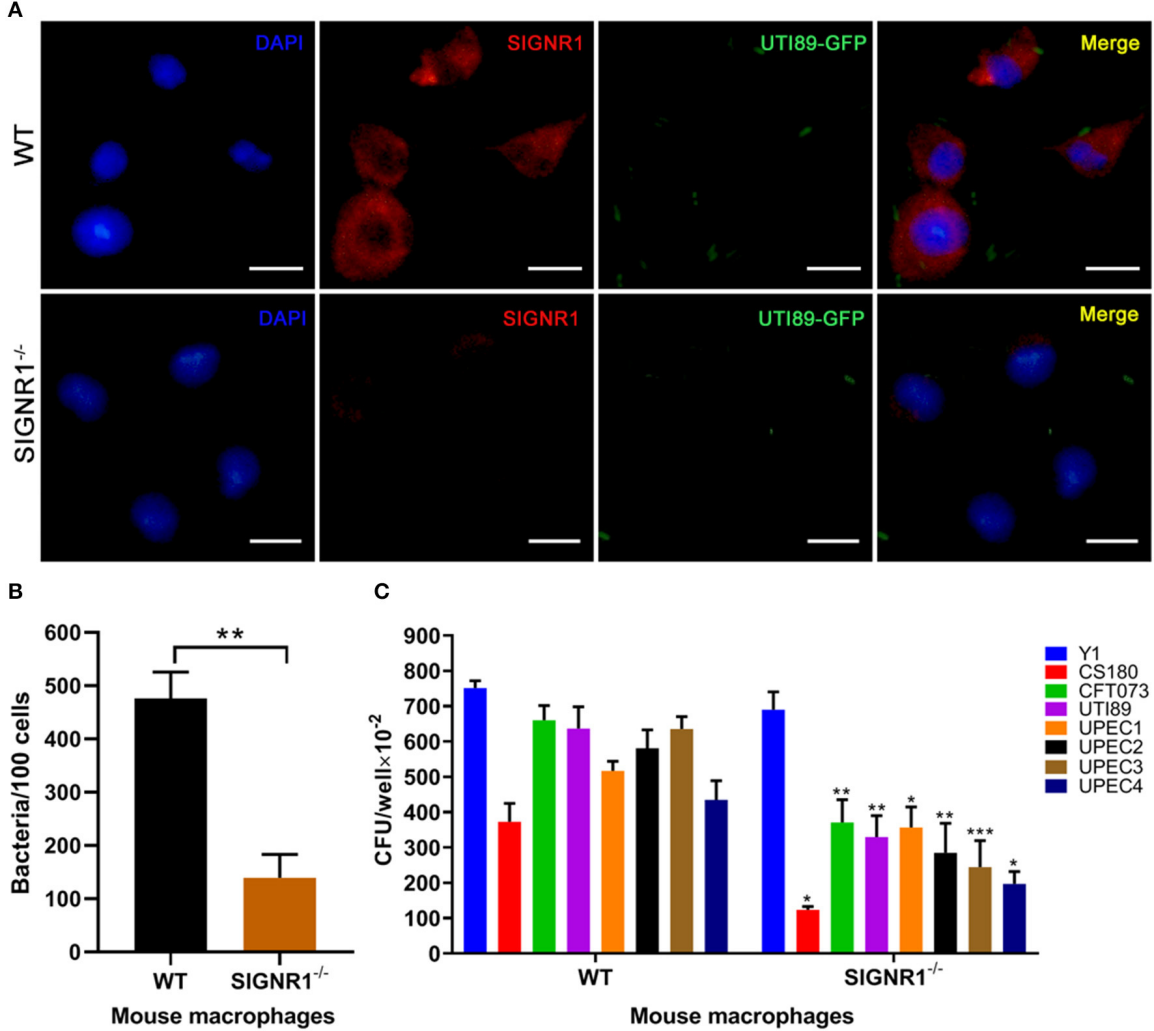
Reduced phagocytosis of uropathogenic *E. coli* by macrophages from SIGNR1^−/−^ mice. **(A)** Immunofluorescence (IF) microscopy of SIGNR1 expression and its interaction with UTI89. Green: UTI89; red: anti-SIGNR1; blue: DAPI. Scale bars ~10 μm in length. **(B)** Quantitation of cell-associated bacteria in macrophages from SIGNR1^−/−^ and WT mice. **(C)** Invasion assays for phagocytosis of UPEC strains by mouse macrophages. *Y. pseudotuberculosis* Y1 and *E. coli* CS180 served as control strains. The results presented were pooled from three independent experiments. Mean ± SEM. **p < 0.01; ***p < 0.001. Mann-Whitney test **(B)**; Two-way ANOVA with Sidak's multiple comparisons test **(C)**.

We have previously shown that human DC-SIGN (hDC-SIGN, hCD209a) is a receptor for *E.coli* K-12 with core lipopolysaccharide (LPS core) (Klena et al., 2005; Zhang et al., 2006). However, we had evidence showing that mouse SIGNR1 (mSIGNR1, mCD209b), not mouse DC-SIGN (mCD209a), serves as a receptor for the LPS core of other Gram-negative bacteria, such as *Yersinia* spp. (Klena et al., 2005; Zhang et al., 2006, 2008; He et al., 2019; Yang et al., 2019). To assess the involvement of mSIGNR1 in the interaction of uropathogenic *E. coli* and macrophages *in vitro*, we evaluated the phagocytic capacity of peritoneal macrophages derived from SIGNR1^−/−^ mice in six UPEC strains. The UTI89 strain carrying a pAcGFP1 plasmid (UTI89-GFP) was used for immunofluorescence analysis. After incubation with UTI89-GFP, peritoneal macrophages from WT mice or SIGNR1^−/−^ mice were stained for SIGNR1, and the distributions of SIGNR1 and bacteria were investigated by immunofluorescence (Figure 1A). Macrophages from WT mice expressed high levels of SIGNR1. The cell-associated bacteria were also quantified, and macrophages from SIGNR1^−/−^ mice showed less cell-associated bacteria than macrophages from WT mice (Figure 1B). Additionally, phagocytosis of UPEC by peritoneal macrophages from SIGNR1^−/−^ mice was significantly reduced compared to that of macrophages from WT mice (Figure 1C). The reduced phagocytosis of SIGNR1^−/−^ macrophages was not attributed to the effect of SIGNR1 absence on macrophage viability or reactive oxygen species (ROS), which can be produced as early as 30 min post-phagocytosis (West et al., 2011) (Figures S1, S2). Taken together, this result indicates that macrophages lacking SIGNR1 exhibit reduced phagocytosis of UPEC strains, suggesting the involvement of SIGNR1 in the interaction between UPEC and macrophages.

### Human DC-SIGN and Murine SIGNR1 Are Receptors for Phagocytosis of UPEC

**Figure 2:**
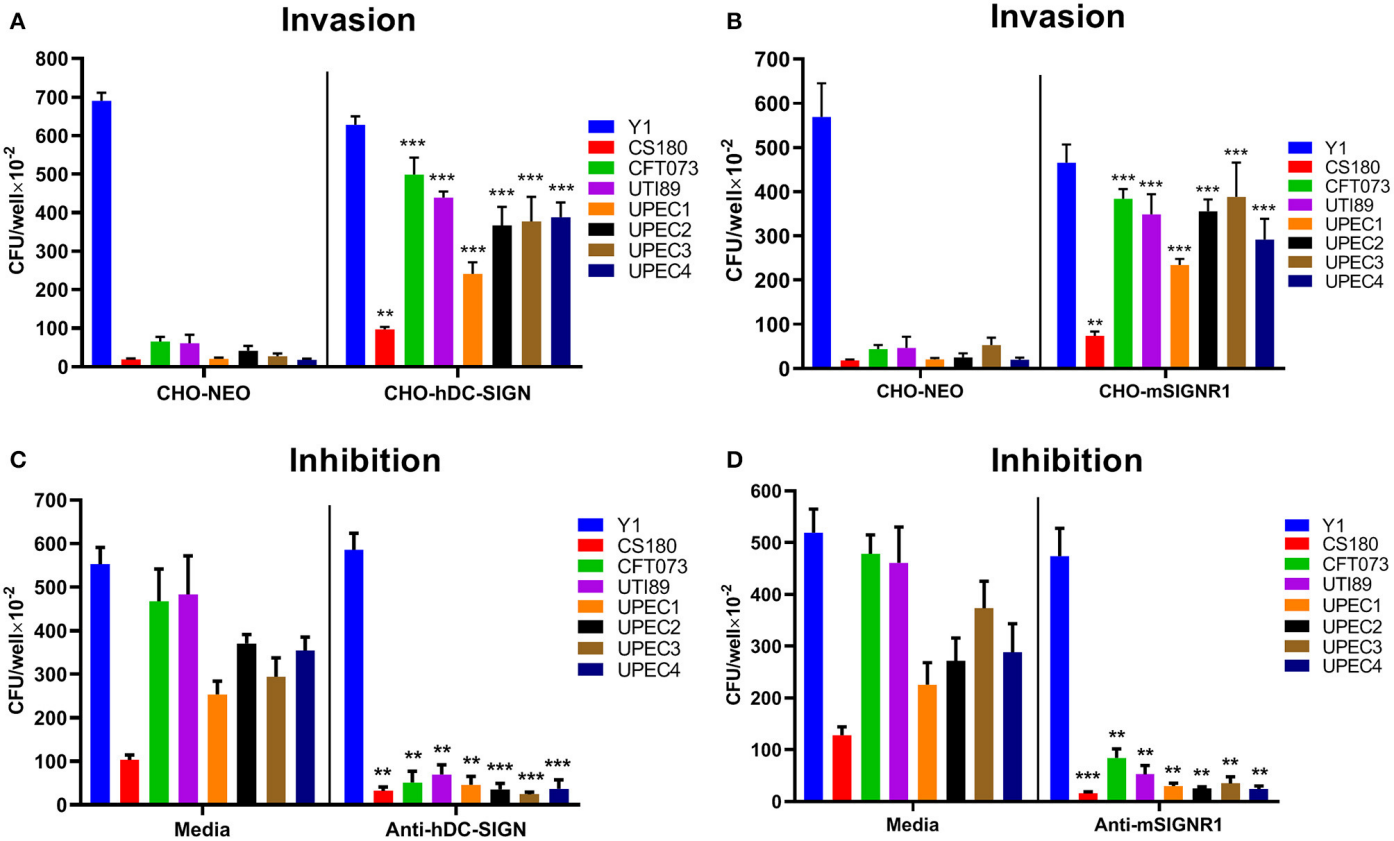
Human DC-SIGN and mouse SIGNR1 are receptors for phagocytosis of UPEC strains. *Y. pseudotuberculosis* Y1 and *E. coli* CS180 were control strains. Phagocytosis of UPEC strains by **(A)** CHO/CHO-hDC-SIGN and **(B)** CHO/CHO-mSIGNR1 cells and inhibition by **(C)** anti-hDC-SIGN or **(D)** anti-mSIGNR1 were conducted as described in Methods. The number of phagocytosed bacteria was determined by counting CFUs recovered following gentamycin treatment. The results presented were pooled from three independent experiments. Mean ± SEM. **p < 0.01; ***p < 0.001. Two-way ANOVA with Sidak's multiple comparisons test.

To investigate whether hDC-SIGN and mSIGNR1 were responsible for the interaction of UPEC with human DCs and mouse macrophages, stably transfected cell lines expressing human DC-SIGN (CHO-hDC-SIGN) and murine SIGNR1 (CHO-mSIGNR1) were tested for their ability to phagocytose UPEC strains. Y1 (cultured at 26°C) and *E. coli* CS180, mediating SIGNR1-independent and SIGNR1-dependent interactions, respectively, were again used as control strains. UPEC strains invaded CHO-hDC-SIGN (Figure 2A) and CHO-mSIGNR1 (Figure 2B) cells more effective than that of CHO-NEO cells, indicating that hDC-SIGN and mSIGNR1 are receptors for the phagocytosis of UPEC strains by epithelial cells and macrophages. Moreover, the interaction between CHO-hDC-SIGN (Figure 2C) or CHO-mSIGNR1 (Figure 2D) and UPEC strains was significantly blocked by the addition of antibodies, suggesting a specific UPEC-CD209s interaction, which promotes bacterial invasion into mouse macrophages. Interestingly, after transformation with a plasmid that expresses O-antigen, phagocytosis of UTI89-O^+^ and CFT073-O^+^ by CHO-hDC-SIGN or CHO-mSIGNR1 cells was significantly reduced (Figure S3). Thus, the epitopes involved in UPEC-CD209s interaction may be shielded by over-expression of O-antigen.

### The Interaction Between UPEC and CD209s Is LPS Core Independent

**Figure 3:**
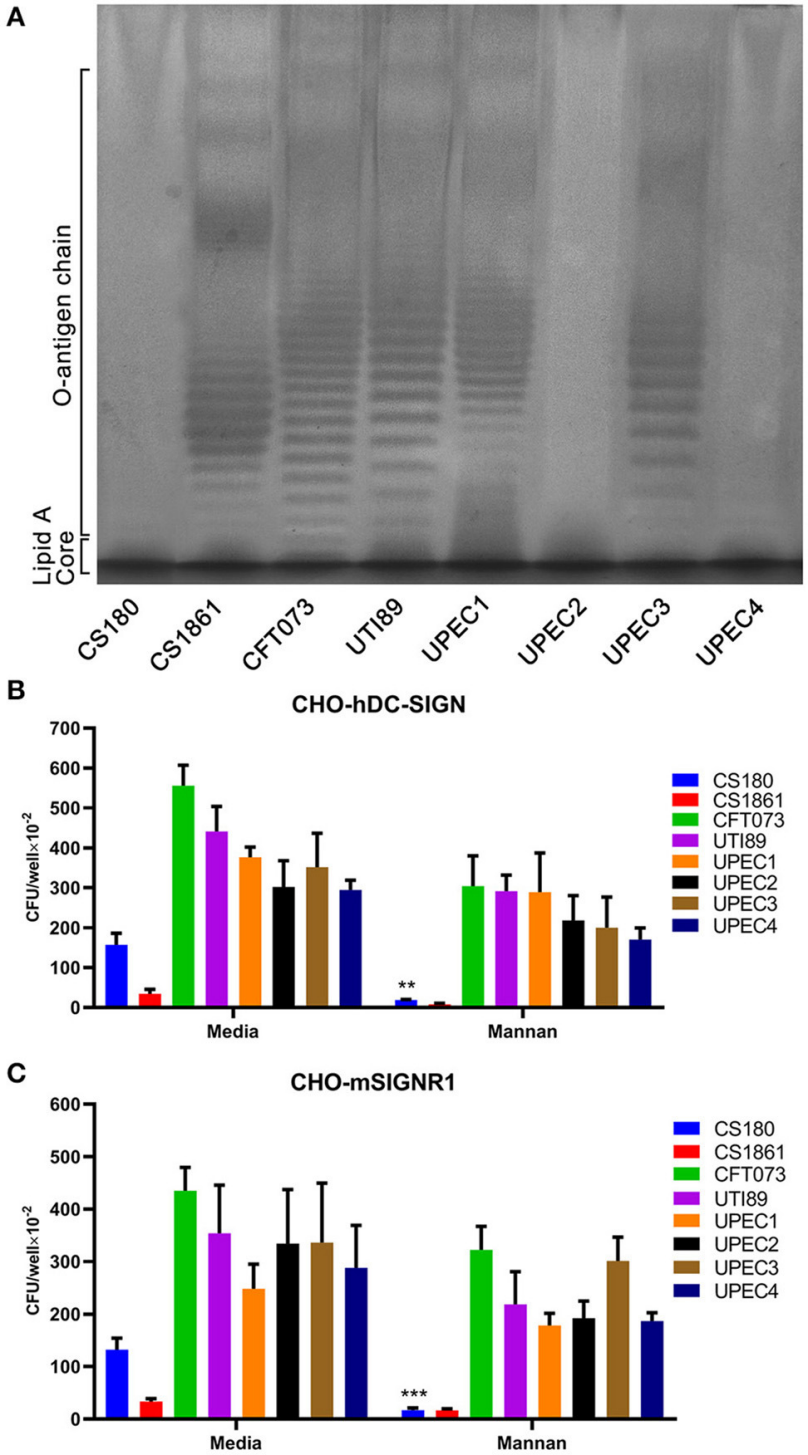
The UPEC-CD209s interaction could not be inhibited by mannan. **(A)** Silver-staining of the LPS of UPEC strains. Controls: *E. coli* strains CS180 and CS1861, which show rough LPS (without O-antigen) and smooth LPS (with O-antigen), respectively. The data presented are representative of three independent experiments. Phagocytosis of UPEC by **(B)** CHO-hDC-SIGN cells and **(C)** CHO-mSIGNR1 cells. The phagocytosis rate was determined by the recovery of bacteria following gentamycin treatment. The data presented were pooled from three independent experiments. Mean ± SEM. **p < 0.01; ***p < 0.001. Two-way ANOVA with Sidak's multiple comparisons test **(B,C)**.

Our recent studies showed that the LPS core of several Gram-negative bacteria, including *Yersinia* spp. and *S*. Typhimurium, exposed by loss of O-antigen, interacts with CD209s (He et al., 2019; Yang et al., 2019; Ye et al., 2019). To determine whether the LPS core of UPEC strains is a ligand for CD209s, we conducted silver staining of LPS from clinical isolates. The isolates UPEC2 and UPEC4 showed no expression of O-antigen, which is in accordance with CS180, a rough strain (Figure 3A). The invasion assays showed that there were no significant differences between these isolates upon interaction with macrophages or CD209s-expressing cell lines (Figures 1, 2), indicating that the interaction between UPEC strains and CD209s may not be mediated by a LPS core-CD209s interaction.

Mannan, which specifically binds mannose-related receptors and LPS core, inhibiting HIV-CD209 or LPS core-CD209s interactions (Geijtenbeek et al., 2000; Zhang et al., 2006), was used for an inhibition assay. Mannan inhibits the interactions between CS180 and CHO-hDC-SIGN or CHO-mSIGNR1 cells, as shown in our previous publication (Klena et al., 2005), but can limitedly inhibit hDC-SIGN- or mSIGNR1-mediated interactions with UPEC strains (Figures 3B,C). We therefore speculate that other sugar residues or LPS core with altered conformation on the surface of UPEC strains may mediate UPEC' interactions with host cells (Zhang et al., 2006; Yang et al., 2015).

### Fewer Bacteria Were Disseminated to the Spleen and Liver in SIGNR1^−/−^ Mice Than in WT Mice

**Figure 4:**
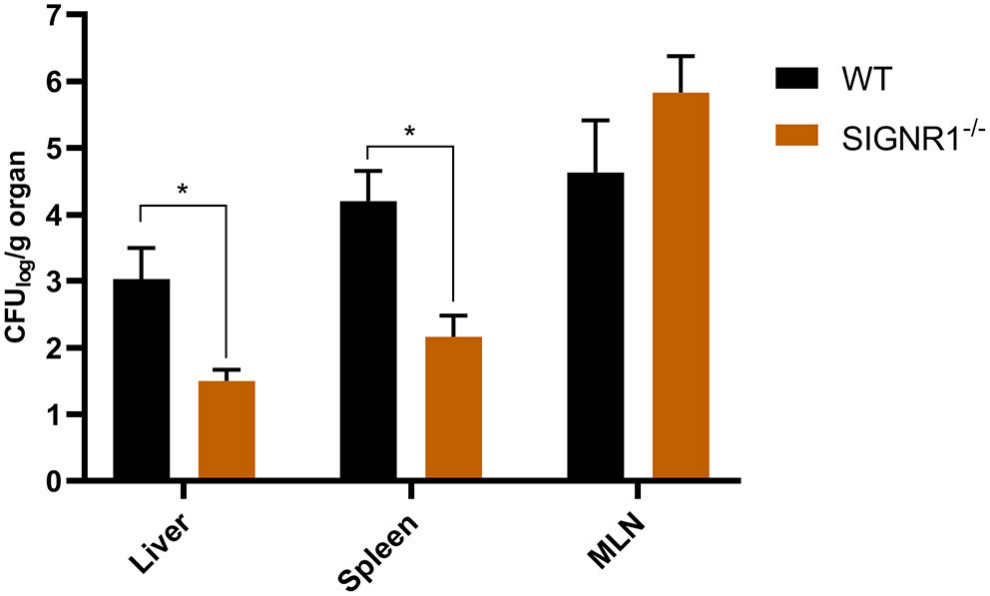
Fewer bacteria were disseminated to livers and spleens in SIGNR1^−/−^ mice than in WT mice. WT mice and SIGNR1^−/−^ mice (*N* = 5 mice/group) were intraperitoneally infected with 10^6^ CFUs of UTI89. Mice were sacrificed at 24 h post-infection, and homogenates of livers, spleens, and mesenteric lymph nodes were plated onto LB plates. The dissemination was determined by CFUs recovered from these organs. The data presented were pooled from three independent experiments. Mean ± SEM. *p < 0.05. Two-way ANOVA with Sidak's multiple comparisons test.

Recent studies showed that *Salmonella* spp. and *Yersinia* spp. could target hDC-SIGN and mSIGNR1 receptors, leading to their dissemination and infection (He et al., 2019; Yang et al., 2019; Ye et al., 2019). We therefore hypothesized that the dissemination of UPEC to livers, spleens, and mesenteric lymph nodes (MLNs) would also be facilitated by this host-pathogen interaction. Therefore, if the interaction could be inhibited, dissemination should be reduced. SIGNR1^−/−^ mice and WT mice were intraperitoneally injected with UTI89 to evaluate UPEC dissemination in the murine host. Lower numbers of UTI89 were isolated from the livers and spleens of SIGNR1^−/−^ mice compared to those of WT mice (Figure 4). Upon infection, both WT and SIGNR1^−/−^ mice showed weight loss and reduced activities, but the amount of weight lost did not statistically differ between the WT and SIGNR1^−/−^ mice at 24 h post-infection (Figure S4). Thus, the decreased dissemination of bacteria in SIGNR1^−/−^ mice may mainly reflect the reduced phagocytosis of UPEC by macrophages.

### SIGNR1^−/−^ Mice Have Impaired Bacterial Clearance Ability Upon Uropathogenic *E. coli* Infection

**Figure 5:**
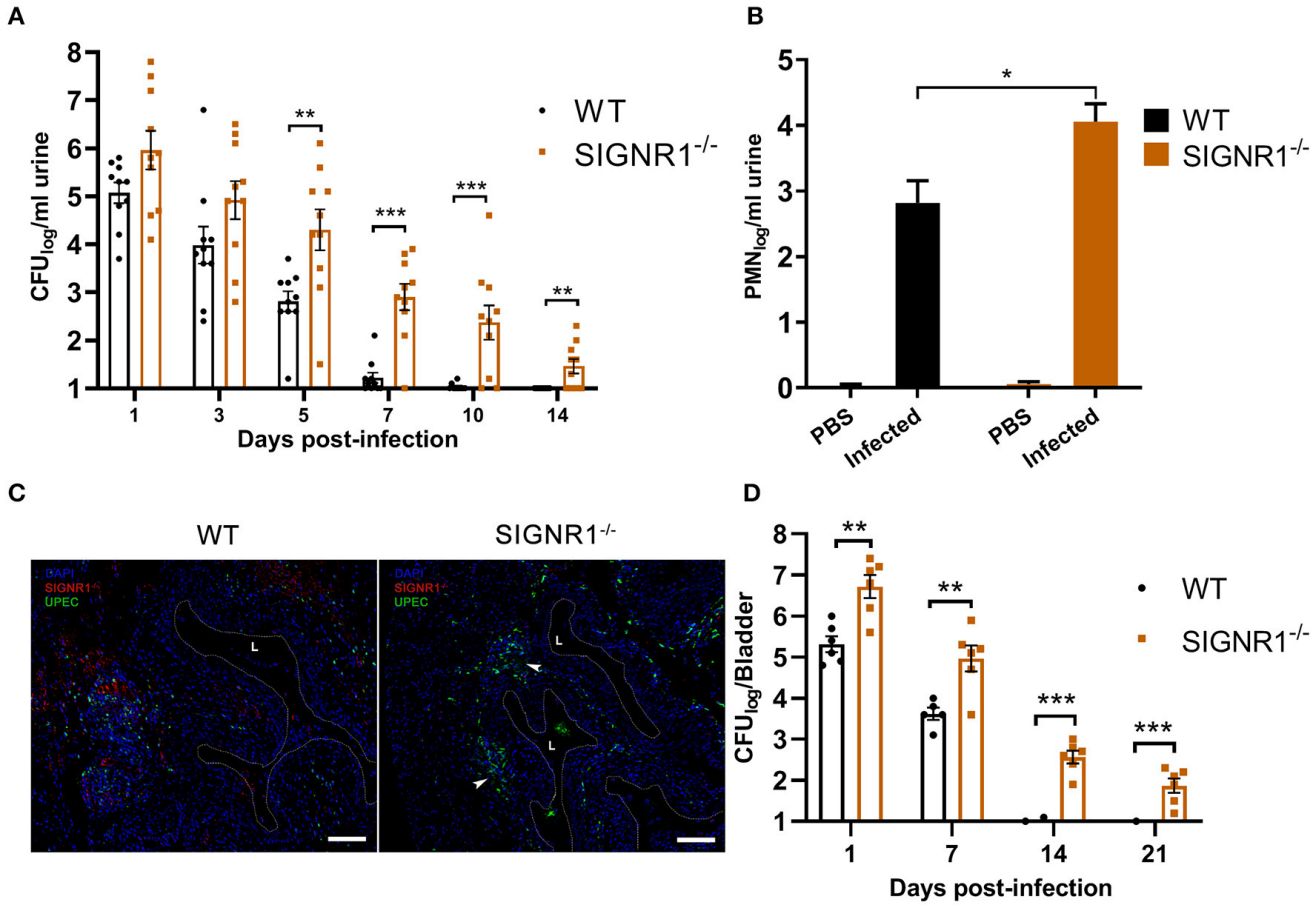
SIGNR1^−/−^ mice had higher bacterial titers in the urine and bladder than WT mice. **(A)** Urine bacterial titers of SIGNR1^−/−^ and WT mice (N = 10 mice/group) time course over 2 weeks post-infection with 10^8^ CFUs of UTI89. Each point represents the value from an individual mouse and the data presents mean ± SEM. **(B)** Hemocytometer counts of polymorphonuclear leukocyte (PMN) levels in the urine of mice 24 h post infection. **(C)** Immunofluorescence of bladders from WT and SIGNR1^−/−^ mice (*N* = 3 mice/group) at 24 h post-infection. Bladder sections were stained for mouse SIGNR1 (red) and UPEC (green) by related monoclonal antibodies, with nuclei counterstained with bis-benzimide (blue). “L” denotes bladder lumen; dashed line indicates approximate location of epithelial surface; arrowheads point to bacterial colonization in the right panel. Scale bars approximate 100 μm in length. Data are representative of three independent experiments. **(D)** Bladder bacterial titers of SIGNR1^−/−^ and WT mice (*N* = 6/group) time course over 3 weeks post-infection with 10^8^ CFUs of UTI89. Each point represents the value from an individual mouse. The data presented were pooled from three independent experiments. Mean ± SEM. *p < 0.05; **p < 0.01; ***p < 0.001. Two-way ANOVA with Sidak's multiple comparisons test **(A.B,D)**.

Murine SIGNR1 is involved in interactions between UPEC strains and host cells *in vitro*. To test whether SIGNR1 is functionally essential during infection with UPEC, we infected SIGNR1^−/−^ and WT mice with cystitis isolate UTI89. After the initial inoculation with 10^8^ CFUs of bacteria, bacterial titers in urine and bladder, as well as histopathological scores, were measured to evaluate the host response to UPEC infection. The SIGNR1^−/−^ mice had higher bacterial titers in urine by day 5 compared to the levels in WT mice (Figure 5A). All the infected WT mice cleared the bacteria within 10 days post-infection, evidenced by sterile urine (Figure 5A). A small population of bacteria could persist in the bladder of mice within or underneath the superficial bladder epithelium, although the urine of these mice is sterile (Chan et al., 2013).

The innate inflammatory response, involving neutrophil recruitment, is activated upon UPEC infection and can resolve the acute phase of bacterial infection (Haraoka et al., 1999). We examined the PMN levels in the urine of infected mice. The urine PMN level in SIGNR1^−/−^ mice was higher than that of WT mice (Figure 5B). Bladders harvested from SIGNR1^−/−^ mice had a higher bacterial burden than those of WT mice at 24 h post-infection (Figure 5C), and no bacteria were detected in mock infection of these mice (Figure S5). Bacterial titers in bladders were also evaluated to determine the persistence of UPEC infections. The UPEC persisted longer in bladders of SIGNR1^−/−^ mice than in those of WT mice. The WT mice exhibited complete clearance of bacteria in the bladder by 14 days post-infection, and SIGNR1^−/−^ mice reached carrier state by 21 days (Figure 5D).

**Figure 6:**
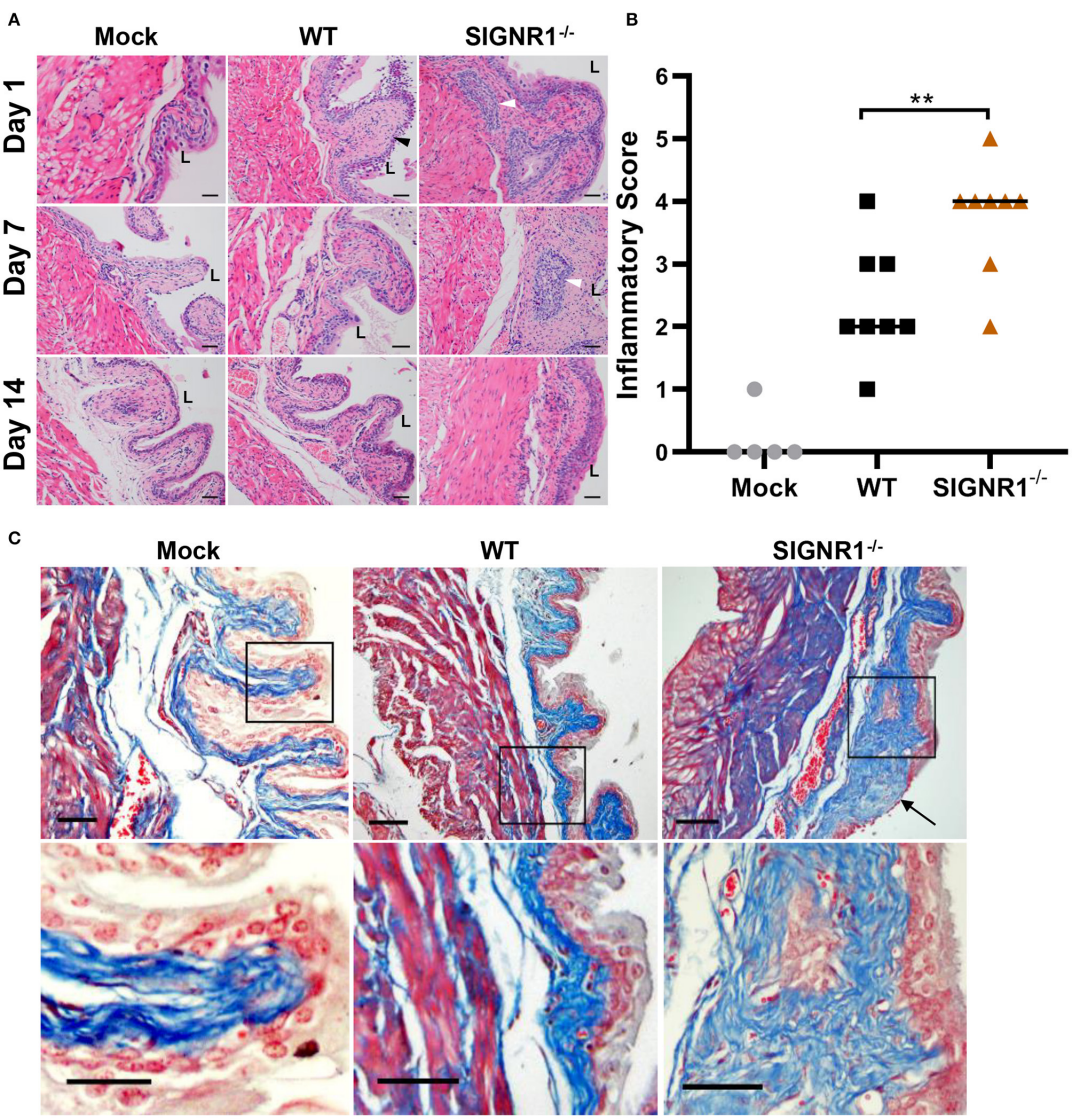
Absence of SIGNR1 increases susceptibility to cystitis caused by UTI89. **(A)** Infected SIGNR1^−/−^ and WT mice (*N* = 8 mice/group) were sacrificed at days 1, 7, and 14 post-infection. Paraffin-embedded, fixed bladder sections were stained with hematoxylin and eosin (HE) and examined by light microscopy. “L” denotes bladder lumen; the black triangles point to exfoliation of epithelial cells; white triangles indicates abscess area. Scale bars ~100 μm in length. **(B)** Hematoxylin and eosin staining of bladder sections from 7 days post-infection mice. Sections were scored from 0 to 5 to quantify the severity of inflammatory lesions, horizontal bars indicate median values **p < 0.01. One-way ANOVA with Holm-Sidak's multiple comparisons test. **(C)** Masson' trichrome staining of bladder sections of 21 days post infection mice (*N* = 5 mice/group). The black boxes in upper panels correspond to the higher magnification images in lower panels. Scale bars ~100 μm in length. The black arrow indicates collagen deposition and epithelial remodeling. “L” denotes bladder lumen.

Histopathological analysis of bladder tissue was performed at different time points on a random subset of mice. Both WT and SIGNR1^−/−^ mice exhibited robust inflammatory responses, including neutrophil infiltration accompanied by edema, urothelial exfoliation, and crypt abscess (Figure 6A). Bladders from SIGNR1^−/−^ demonstrated lesions of inflammation with more abundant neutrophil infiltration and crypt abscess than those observed in WT mice throughout the experimental period (Figure 6A). Moreover, WT mice appeared to resolve the acute phase of infection by 2 weeks post-infection. The inflammatory scores obtained from histopathological analysis showed that SIGNR1^−/−^ mice suffered more severe inflammatory lesions than WT mice did (Figure 6B). The observations from Masson' trichrome staining revealed epithelial lesions in the bladders of infected mice by 3 weeks post-infection. Urothelial hyperplasia and remodeling were observed in bladders of SIGNR1^−/−^ mice, while WT mouse bladders showed almost normal structure (Figure 6C). These results indicate that the mice lacking SIGNR1 have an impaired ability to clear bacteria.

### No Difference in Cytokine Production in Bladders of SIGNR1^−/−^ or WT Mice in Response to UPEC

**Figure 7:**
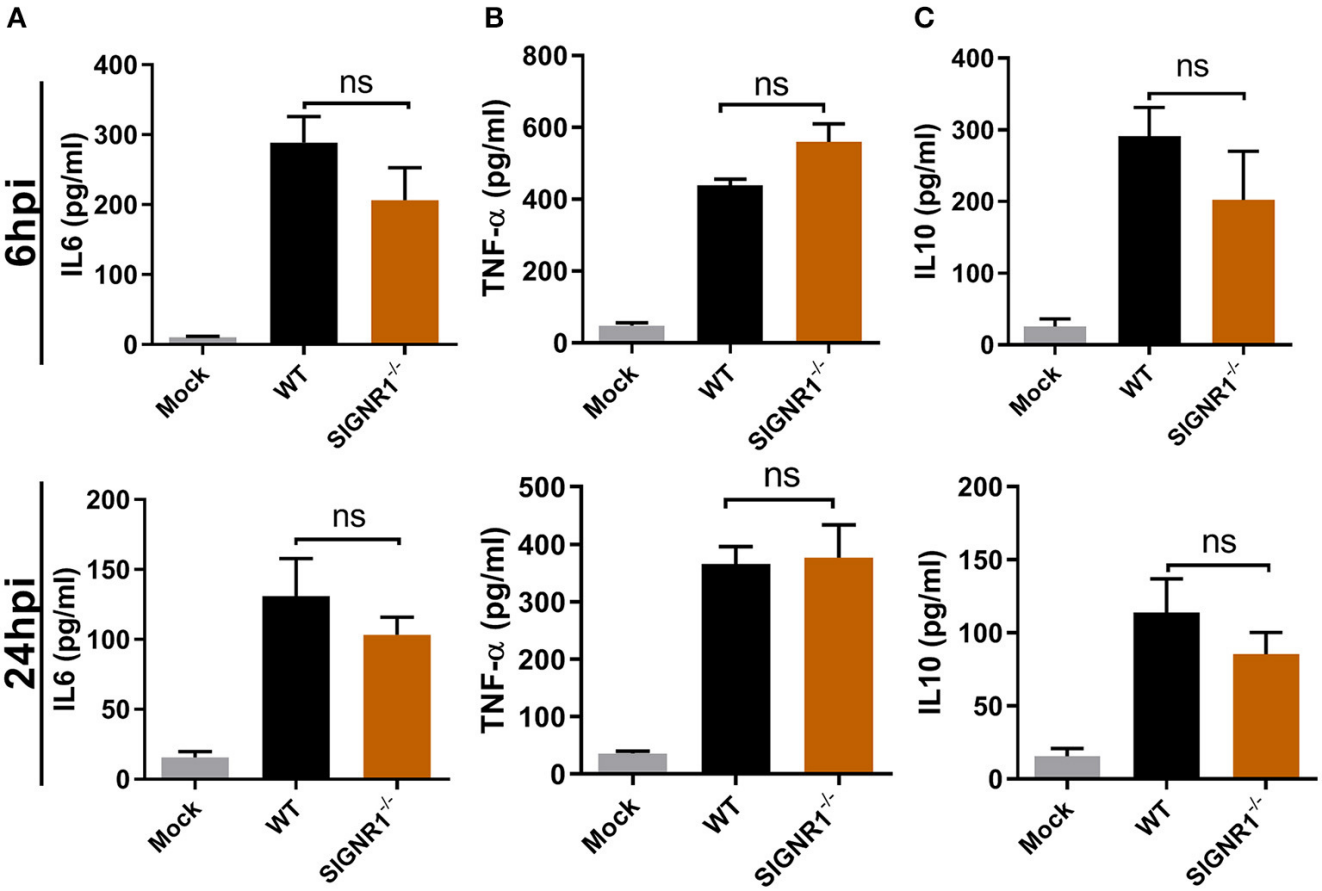
No differences in cytokine IL6, TNF-α, or IL10 production in bladders of SIGNR1^−/−^ or WT mice upon UPEC infection. Bladders of WT and SIGNR1^−/−^ mice (*N* = 5 mice/group) at 6 and 24 h post-infection with PBS or 10^8^ CFUs of UTI89 were harvested for cytokine **(A)** IL6, **(B)** TNF-α, and **(C)** IL10 assays in ELISA. The data presented were pooled from three independent experiments. Mean ± SEM. ns, no significance. One-way ANOVA with Holm-Sidak's multiple comparisons test.

To better understand the underlying differences in inflammatory response to UPEC infection in the SIGNR1^−/−^ and WT mice, we studied the cytokine profile of bladder tissues in both mice groups during *E. coli* infection at the expected time frame for immune response. As expected, a strong proinflammatory response, demonstrated by drastic cytokine expression, including IL-6 and TNF-α, was observed early upon infection (Figures 7A,B). Interestingly, a major inhibitor of proinflammatory immune responses, IL-10, exhibited high levels of expression parallel to the expression levels of proinflammatory cytokines (Figure 7C). However, we found that there were no differences between the SIGNR1^−/−^ and WT mice in the local IL-10 response, nor in the levels of IL-6 and TNF-α. The IL-10 level in SIGNR1^−/−^ mice was slightly lower than that of WT mice, indicating that severe inflammation of SIGNR1^−/−^ may partly be related to IL-10 expression. Based on this cytokine profile, we could not determine a role for SIGNR1 in the induction of cytokine production to modulate the inflammatory response and the concomitant cellular infiltration upon infection with UPEC.

## Discussion

Urinary tract infections (UTIs) caused by uropathogenic *E. coli* are characterized by a high incidence of recurrence. About half of recurrent UTIs are attributed to the same UPEC strain that caused the initial infection (Harrabi, 2012; Silverman et al., 2013), indicating that individuals with UTIs fail to mount an efficient immune response that protects against subsequent infections in the urinary tract. However, the molecular mechanism of how UPEC cause persistent infection remains largely unclear. In a recent study, we demonstrated that *S*. Typhimurium targets DC-SIGN (CD209s) expressed by APCs to promote host dissemination and infection in an animal model, which may provide an explanation of a persistent infection, such as carried by “Typhoid Mary.” The identification of the molecular mechanism of persistent bacterial infection, such as *Helicobacter pylori*-associated chronic gastritis and *Mycobacterium tuberculosis*-associated tuberculosis, would facilitate treatment of infections. These intracellular pathogens all share the ability to interact with CD209s for attachment and invasion of host cells (Tailleux et al., 2003; Driessen et al., 2009; Carroll et al., 2010; Miszczyk et al., 2012; Ye et al., 2019). Moreover, studies by Helaine *et al*. demonstrated that *S. typhimurium* could form intracellular persisters in macrophages that serve as reservoirs of bacterial persistence. In an UTI model, macrophages comprise nearly 40% of all APCs in the bladder immune cell compartment of mice (Mora-Bau et al., 2015), suggesting that macrophages may play important roles in local immune surveillance. Phagocytosis is a prominent feature of the macrophages' antibacterial host response and is mediated by pattern recognition receptors (PRRs). Hosts can counteract invading bacterial pathogens utilizing their defensive weapons of APCs, including DCs and macrophages. The interactions between host macrophages and uropathogens are considered crucial to initiate an efficient immune response that defends against invading bacteria in UTIs (Mora-Bau et al., 2015; Owusu-Boaitey et al., 2016; Lacerda Mariano and Ingersoll, 2018).

This study demonstrates that human DC-SIGN and murine SIGNR1 are receptors for recognition and phagocytosis of UPEC strains by CD209s-expressing macrophages and transfectants. The UPEC-CD209s interaction can be significantly inhibited by the addition of an anti-CD209s antibody, indicating that CD209s are receptors for UPEC. However, the mSIGNR1- or hDC-SIGN-mediated interactions was only limitedly inhibited by mannan in this study, which shows discrepancy to our results in previous studies (Zhang et al., 2006; Ye et al., 2019). Two out of four clinical UPEC isolates were rough strains without O-antigen, but showed no differences in their abilities to invade host cells, suggesting that the UPEC-CD209s interaction was LPS core-independent. Interestingly, after transfection with O-antigen-expressing plasmid, the UPEC strain UTI89 exhibited significantly decreased phagocytosis by CD209-expressing transfectants, indicating that the over-expression of O-antigen may shield the interface of the UPEC-CD209s interaction. The type 1 pilus-associated adhesin FimH is the major facilitator of UPEC entry into host cells, and has been reported to bind a wide range of mannose-containing receptors (Leusch et al., 1991; Kukkonen et al., 1993; Eto et al., 2007; Mossman et al., 2008; Ielasi et al., 2016). We therefore examined the possible binding of FimH to mSIGNR1 with the recombinant FimH protein. The result indicates that the FimH can inhibit interactions between the UTI89 strain and CHO/CHO-mSIGNR1 cells; however, the FimH could bind to both CHO and CHO-mSIGNR1 cells, suggesting that the FimH may not specific ligand for the SIGNR1 receptor (Figure S6). Additionally, the sialic acids of polysaccharide capsules play crucial roles in bacterial resistance to phagocytosis and serum killing, as well as the formation of IBCs (Buckles et al., 2009; Anderson et al., 2010; Goller and Seed, 2010). Given the affinity of CD209s to carbohydrate chains exposed on several Gram-negative pathogens, further studies are needed to specify the ligands on UPEC.

SIGNR1 (CD209b) is a mouse homolog of the human DC-SIGN receptor that is reported to interact with several bacterial species (Tailleux et al., 2003; Lanoue et al., 2004; Yang et al., 2019; Ye et al., 2019), but its functional role *in vivo* in UTIs has not been well-defined. We examined SIGNR1-deficient (SIGNR1^−/−^) mice in a UTI model. We demonstrate that in SIGNR1^−/−^ mice, the bacterial burden in the bladder and the intensity of inflammatory responses, were significantly higher than those of WT mice over time post-infection. The difference in the inflammatory response upon UPEC infection between SIGNR1^−/−^ and WT mice is not due to changes in the expression levels of IL-6, TNF-α, or IL-10, but is consistent with the observed reduction of the phagocytic ability of SIGNR1^−/−^ macrophages. Nevertheless, it appears that the IL-10 level in SIGNR1^−/−^ mice was slightly lower than that of WT mice, suggesting that severe inflammation of SIGNR1^−/−^ may partly be related to IL-10, which is characterized as an immunosuppressive cytokine.

The C-type lectin receptors (CLRs) are involved in modulatory processes of immunity by cross-talk with other PRRs, such as Toll-like receptors (TLRs). Studies by Geijtenbeek et al. showed that the binding of DC-SIGN with ManLAM derived from *Mycobacterium tuberculosis* affects intracellular signaling pathways through communication with TLRs, thus modulating cytokine production involved in inflammatory responses (Geijtenbeek et al., 2003; Gringhuis et al., 2007, 2009). Because of its broad specificity for mannose and/or fucose-terminated glycan, DC-SIGN can mediate the interaction between host cells and numerous pathogens (Ehlers, 2010; Miszczyk et al., 2012; He et al., 2019; Ye et al., 2019). In our recent work, the SIGNR1^−/−^ mice were more susceptible to *Y. pestis* infection compared to WT mice (Yang et al., 2019), and this difference may reflect the multifunctional properties of this receptor in the host immune system. The complement system plays a significant role in hosts' anti-infection processes. Kang *et al*. found involvement of SIGNR1 in the complement fixation pathway by interaction with C1q (Kang et al., 2006). Overall, experiments with knockout mice are required to better understand the in vivo function of SIGNR1. However, there are potential limitations of this approach to study bacteria-host cell interactions, and this strategy may only work if SIGNR1 is the only, or the main receptor for pathogens. Unfortunately, many pathogens do not depend on only one receptor in their interactions with host cells.

In summary, this is the first demonstration that human DC-SIGN and mouse SIGNR1 are cellular receptors for recognition and phagocytosis of uropathogenic *E. coli* by host cells. Murine SIGNR1 contributes to clearance of UPEC in an UTI model. However, the specific UPEC molecules that bind to CD209s were not identified. We also observed that over-expression of O-antigen significantly inhibited the UPEC-CD209s interactions. Further investigations are needed to determine the corresponding UPEC ligands, as we did previously for the identification of the carbohydrate LPS core from many Gram-negative bacteria (Klena et al., 2005; Zhang et al., 2006, 2008; Yang et al., 2015, 2019; He et al., 2019; Ye et al., 2019). Several studies showed that in vivo targeting of CD209s drastically enhances antigen presentation as well as persistent CD8+/CD4+ T-cell-mediated protective immunity against intracellular pathogens (Singh et al., 2009; Unger et al., 2012; Hesse et al., 2013; Schetters et al., 2018). Thus, given the protective role of SIGNR1 in UTIs, the interaction between UPEC and CD209s should be targeted in treatment strategies to prevent persistent UTIs.

## Data Availability Statement

The datasets generated for this study are available on request to the corresponding author.

## Ethics Statement

The studies involving human participants were reviewed and approved by Medical Ethics Committee of Tongji Hospital, Tongji Medical College, Huazhong University of Science and Technology. The patients/participants provided their written informed consent to participate in this study. The animal study was reviewed and approved by Institutional Animal Care and Use Committees and Institutional Review Board of Tongji Hospital, Tongji Medical College, Huazhong University of Science and Technology.

## Author Contributions

TC, HCa, and YZ designed and conceived the experiments. YZ, SZha, YH, and BW performed the experiments. YZ, YH, CY, YX, QL, and WL analyzed the data. YL, LJ, and SZhu contributed reagents, materials, and analytical tools. YZ, HCh, WC, ZS, and TC wrote and reviewed the manuscript.

### Conflict of Interest

The authors declare that the research was conducted in the absence of any commercial or financial relationships that could be construed as a potential conflict of interest.
