# Supplementary material for: Murine SIGNR1 (CD209b) Contributes to the Clearance of Uropathogenic Escherichia coli During Urinary Tract Infections
Source: Front Cell Infect Microbiol. 2020 Jan 10;9:457. doi: 10.3389/fcimb.2019.00457 (PMC6965063; doi:10.3389/fcimb.2019.00457)
Supplement: Supplementary file 1 [file Table_1.DOC]

**Supplementary material**

**Materials and Methods**

**Cell viability assays**

The effect of UPEC on macrophage viability was assessed using the (3-(4,5-dimethylthiazol-2-yl))-2,5diphenyltetrazolium bromide (MTT) assay, as previously described (Sweet and Hume, 1996). The macrophages derived from WT and SIGNR1^-/-^ mice were plated in 96-well plate at a concentration of 1×10^4^ cells/well and infected with or without 5×10^4^ CFUs of UTI89. 2 h post-infection, macrophages were treated with MTT (Signalway Antibody Co., Ltd, Maryland, USA) for 4 h according to the manufacturer’s instructions. The blue formazan precipitate was dissolved in dimethyl sulfoxide (DMSO) and absorbance at 570 nm was measured. Cell viability levels were determined by comparing the absorbance of infected group with that of uninfected group (defined as 100%).

**Binding of FimH to epithelial cells**

Recombinant *E.coli* Type 1 fimbriae D-mannose specific adhesion (His-tagged FimH) (CUSABIO inc., Wuhan, China) was incubated with CHO and CHO-mSIGNR1 cells at 37ºC for 1 h as described (Sheikh et al., 2017). Unbound FimH was removed by washing with culture media, and the cells were were fixed in cold methanol for 10 min and incubated for 1 h in 1% BSA (Sigma-Aldrich, MO, USA) in PBS. The cells were then incubated for 1 h at room temperature with a primary rabbit IgG antibody (CUSABIO inc., Wuhan, China) directed against His-FimH. Primary antibodies were detected with a goat anti–rabbit IgG-conjugated with Alexa Fluor 488 (Invitrogen, CA, USA).

**Results**

**
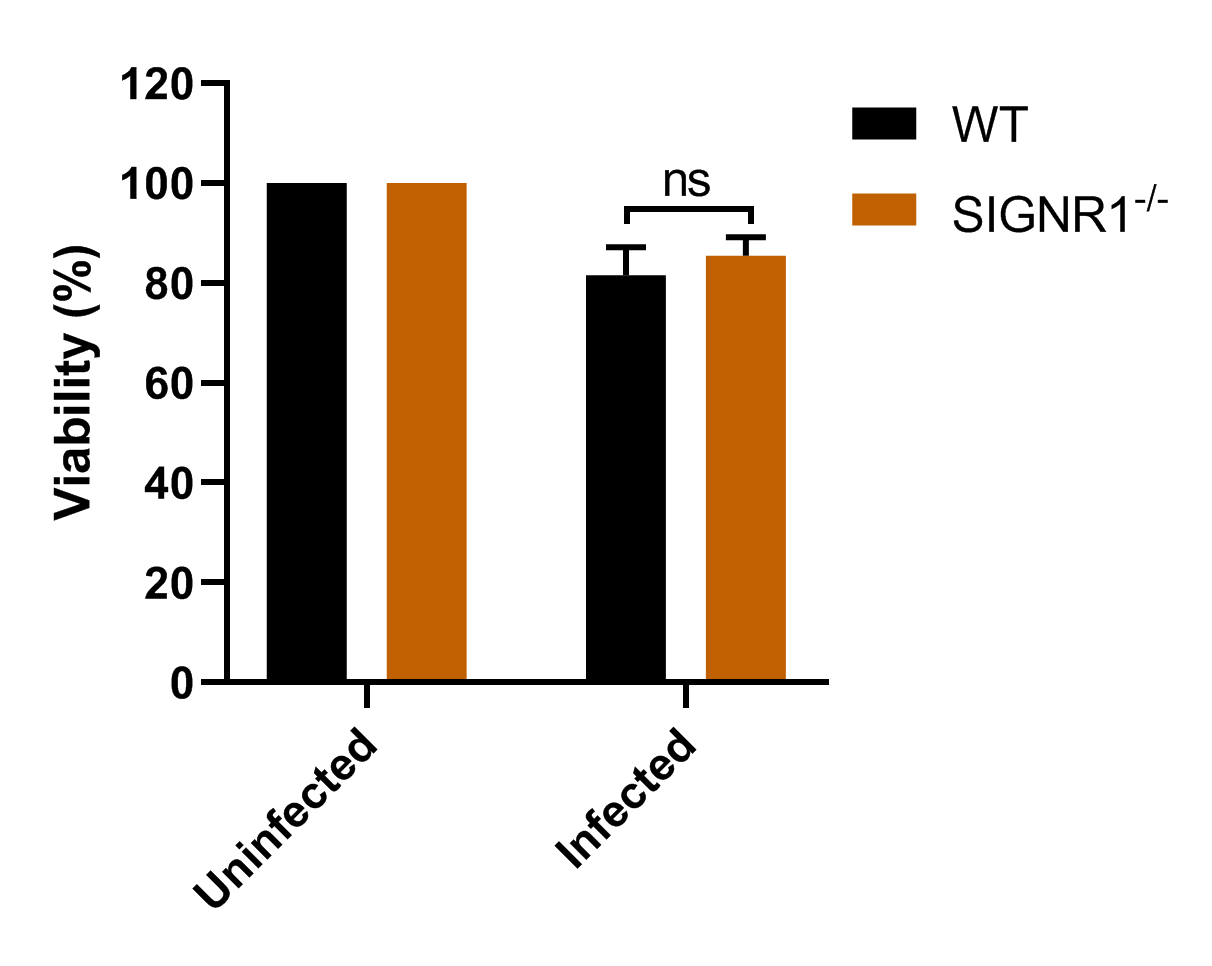
**

**Figure S1** Macrophages derived from WT and SIGNR1^-/-^ mice showed no difference in cell viability at 2 h post-infection with UTI89. Macrophage viability was determined by MTT assay. The data presented were pooled from three independent experiments. Mean ± SEM. ns, no significance. Two-way ANOVA with Sidak’s multiple comparisons test.

**
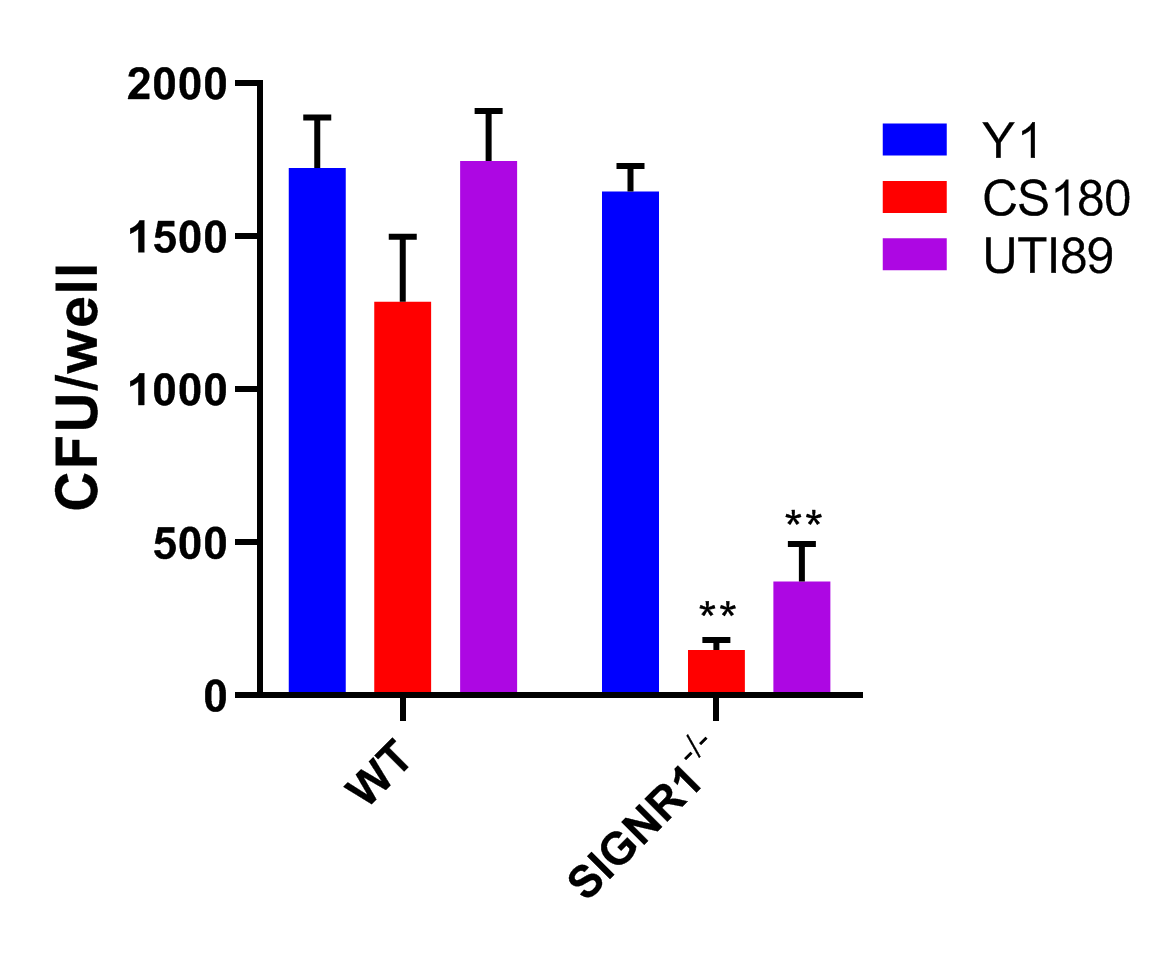
**

**Figure S2** Macrophages derived from SIGNR1^-/-^ mice showed significantly reduced phagocytosis after 10 min incubation with UPEC. *Y. pseudotuberculosis* Y1 cultured at 26ºC and *E.coli* CS180 were used as control strains. The data presented were pooled from three independent experiments. Mean ± SEM. ns, no significance. Two-way ANOVA with Sidak’s multiple comparisons test.


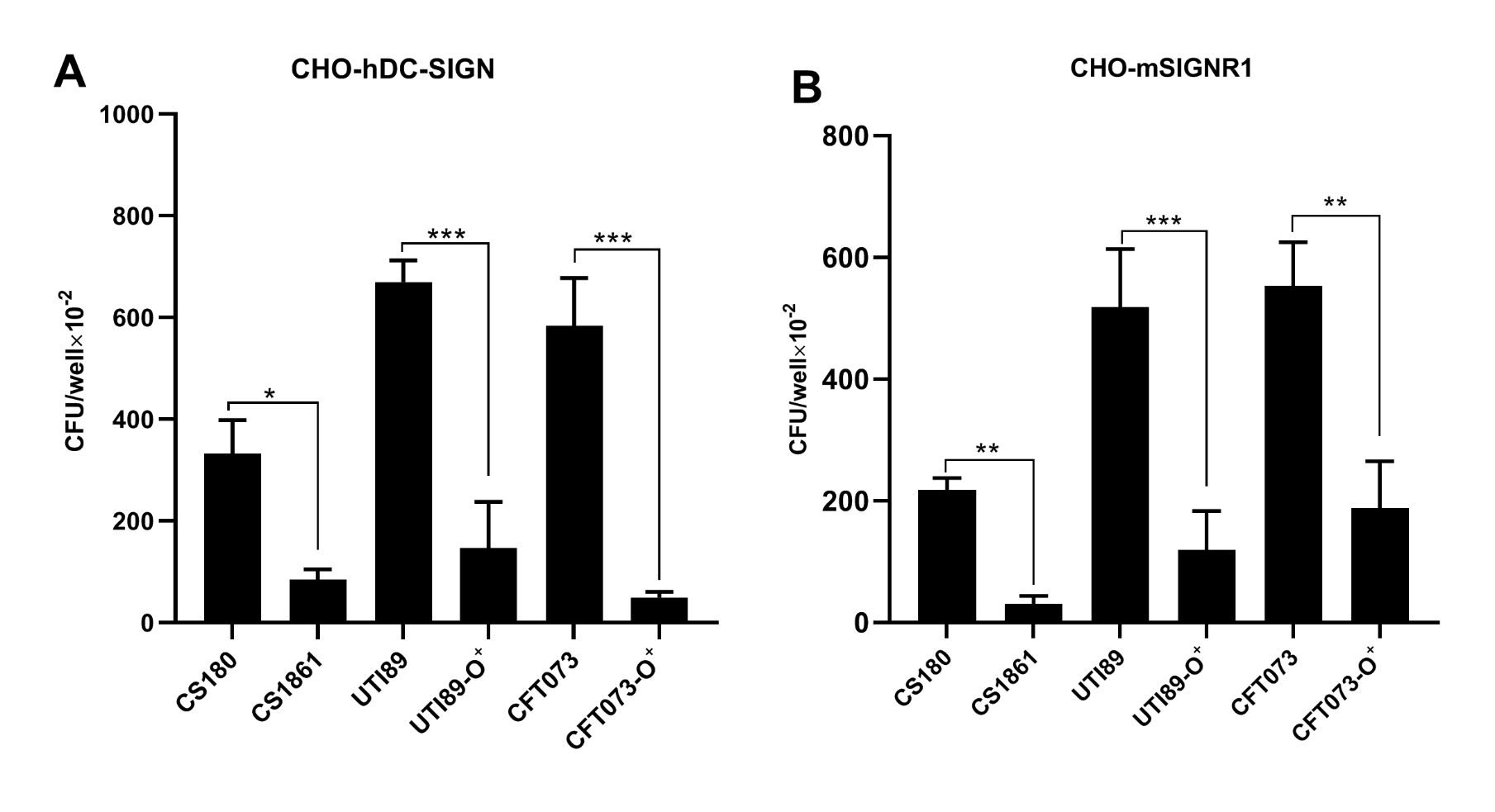


**Figure S3** Over-expression of O-antigen inhibited the interaction between UPEC and CD209s. UTI89 and CFT073 expressing *Y. enterocolitica* O-antigen. *E. coli* CS180 and CS1861, which shows rough LPS (without O-antigen) and smooth LPS (with O-antigen), respectively, were used as control strains. Phagocytosis of **(A)** CHO-hDC-SIGN and **(B)** CHO-mSIGNR1 was determined by CFUs recovered from gentamycin treatment. The data presented were pooled from three independent experiments. Mean ± SEM. **p*<0.05; ***p*<0.01; ****p*<0.001. One-way ANOVA with Holm-Sidak’s multiple comparisons test.

**
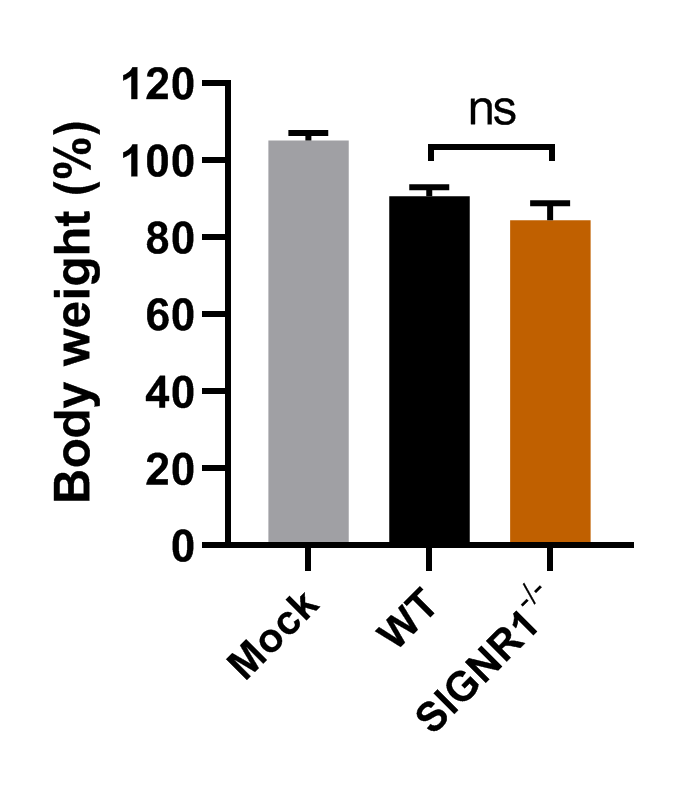
**

**Figure S4** WT and SIGNR1^-/-^ mice showed no difference in weight loss at 24 h post-infection. Mice were intraperitoneally challenged with PBS or 10^6^ CFUs of UTI89. N=5 mice/group. The data presented were pooled from three independent experiments. Mean ± SEM. ns, no significance. One-way ANOVA with Holm-Sidak’s multiple comparisons test.

**
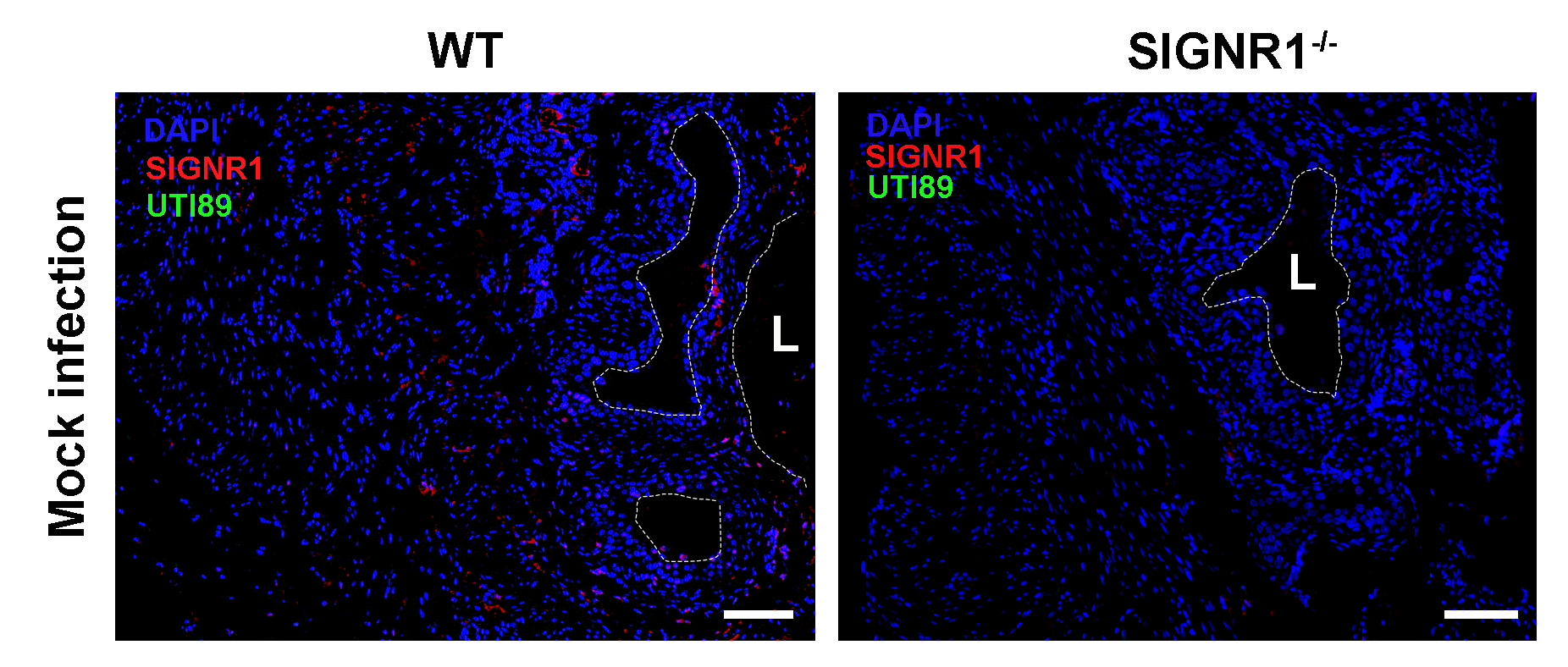
Figure S5** Immunofluorescence of bladders from mock infection (PBS) of WT and SIGNR1^-/-^ mice (N=3 mice/group) at 24 h post-infection. Bladder sections were stained for mouse SIGNR1 (red) and UPEC (green) by related monoclonal antibodies, with nuclei counterstained with bis-benzimide (blue). ‘‘L’’ denotes bladder lumen; dashed line indicates approximate location of epithelial surface; Scale bars approximate 100 μm in length. Data are representative of three independent experiments.


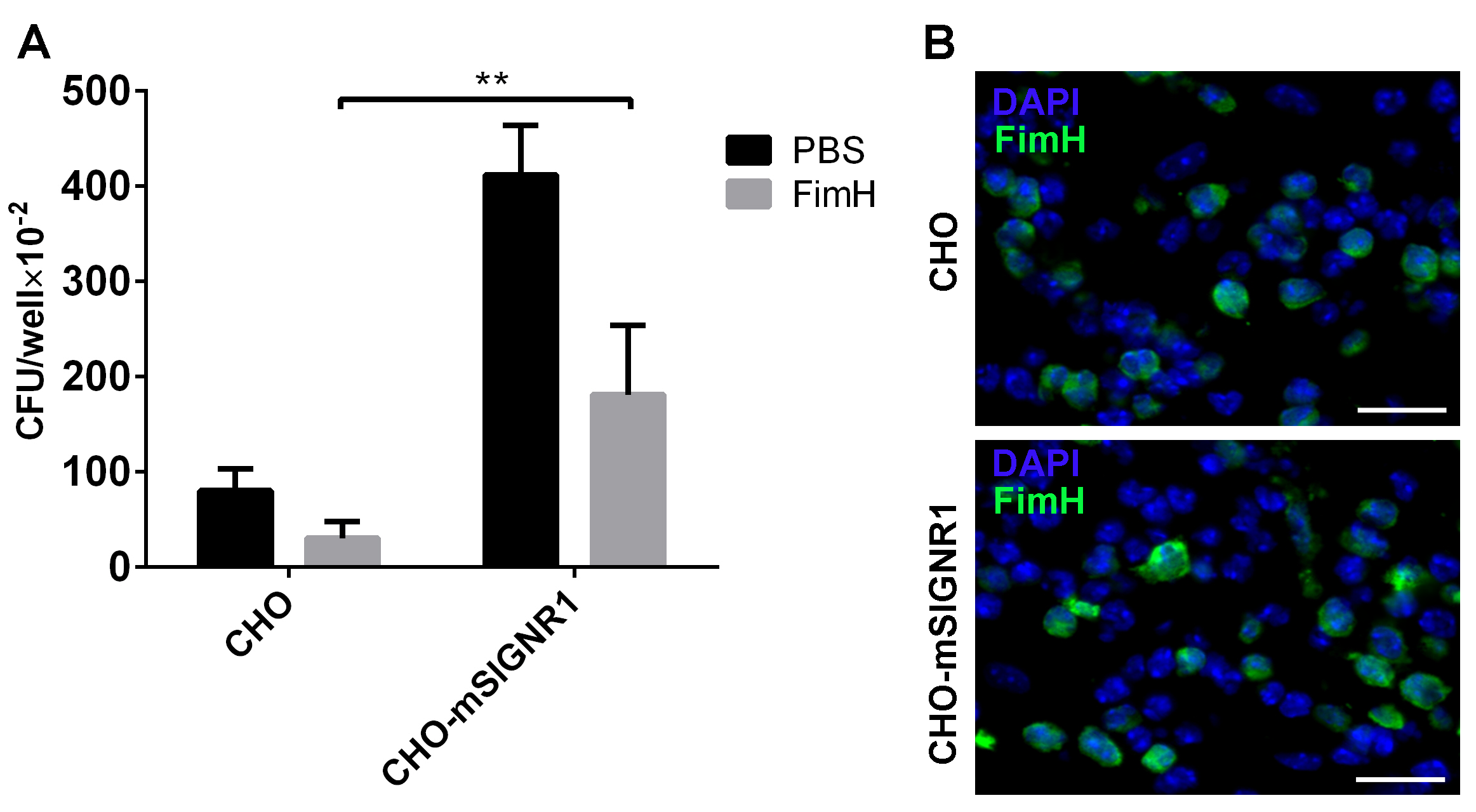


**Figure S6** Exogenous FimH protein inhibits interaction between UPEC and epithelial cells. **(A)** Cells were pretreated with or without FimH protein (5μg/ml) before incubation with UTI89. The data presented were pooled from three independent experiments. Mean ± SEM. **p<0.01. Two-way ANOVA with Sidak’s multiple comparisons test. **(B)** CHO and CHO-mSIGNR1 cells were incubated with 10μg His-FimH for 1 h. The binding of His-FimH to epithelial cells was detected using immunofluorescence. The data were representative of three independent experiments. Scale bar approximate 20μm in length.

**References**

Sheikh, A., Rashu, R., Begum, Y.A., Kuhlman, F.M., Ciorba, M.A., Hultgren, S.J., et al. (2017). Highly conserved type 1 pili promote enterotoxigenic *E. coli* pathogen-host interactions. *PLoS Negl Trop Dis* 11(5)**,** e0005586. doi: 10.1371/journal.pntd.0005586.

Sweet, M.J., and Hume, D.A. (1996). Bacterial lipopolysaccharide confers resistance to G418, doxorubicin, and taxol in the murine macrophage cell line, RAW264. *J Leukoc Biol* 59(2)**,** 280-286. doi: 10.1002/jlb.59.2.280.
